# Dysregulated Autophagy Contributes to Podocyte Damage in Fabry’s Disease

**DOI:** 10.1371/journal.pone.0063506

**Published:** 2013-05-17

**Authors:** Max C. Liebau, Fabian Braun, Katja Höpker, Claudia Weitbrecht, Valerie Bartels, Roman-Ulrich Müller, Susanne Brodesser, Moin A. Saleem, Thomas Benzing, Bernhard Schermer, Markus Cybulla, Christine E. Kurschat

**Affiliations:** 1 Department 2 of Internal Medicine and Center for Molecular Medicine Cologne, University of Cologne, Cologne, Germany; 2 Department of Pediatrics, University of Cologne, Cologne, Germany; 3 Institute for Medical Microbiology, Immunology, and Hygiene, University of Cologne, Cologne, Germany; 4 Cologne Excellence Cluster on Cellular Stress Responses in Aging-Associated Diseases, University of Cologne, Cologne, Germany; 5 Systems Biology of Ageing Cologne, University of Cologne, Cologne, Germany; 6 Academic and Children’s Renal Unit, School of Clinical Sciences, University of Bristol, Southmead Hospital, Bristol, United Kingdom; 7 Renal Division, University Hospital Freiburg, Freiburg, Germany; Institut National de la Santé et de la Recherche Médicale, France

## Abstract

Fabry’s disease results from an inborn error of glycosphingolipid metabolism that is due to deficiency of the lysosomal hydrolase α-galactosidase A. This X-linked defect results in the accumulation of enzyme substrates with terminally α-glycosidically bound galactose, mainly the neutral glycosphingolipid Globotriaosylceramide (Gb3) in various tissues, including the kidneys. Although end-stage renal disease is one of the most common causes of death in hemizygous males with Fabry’s disease, the pathophysiology leading to proteinuria, hematuria, hypertension, and kidney failure is not well understood. Histological studies suggest that the accumulation of Gb3 in podocytes plays an important role in the pathogenesis of glomerular damage. However, due to the lack of appropriate animal or cellular models, podocyte damage in Fabry’s disease could not be directly studied yet. As murine models are insufficient, a human model is needed. Here, we developed a human podocyte model of Fabry’s disease by combining RNA interference technology with lentiviral transduction of human podocytes. Knockdown of α-galactosidase A expression resulted in diminished enzymatic activity and slowly progressive accumulation of intracellular Gb3. Interestingly, these changes were accompanied by an increase in autophagosomes as indicated by an increased abundance of LC3-II and a loss of mTOR kinase activity, a negative regulator of the autophagic machinery. These data suggest that dysregulated autophagy in α-galactosidase A-deficient podocytes may be the result of deficient mTOR kinase activity. This finding links the lysosomal enzymatic defect in Fabry’s disease to deregulated autophagy pathways and provides a promising new direction for further studies on the pathomechanism of glomerular injury in Fabry patients.

## Introduction

Fabry’s disease is a lysosomal storage disorder resulting from an inborn error of glycosphingolipid metabolism [1]. While it has been stated for a long time that Fabry’s disease is rare, occurring with an incidence of 1∶117.000, recent data from newborn screenings in Italy and Taiwan suggest that it is indeed much more common (∼1∶3,100 and 1∶1,600, respectively) [2], [3]. In Fabry’s disease a mutation in the gene encoding α-galactosidase A (α-Gal A) on the X chromosome (*GLA* gene) leads to deficiency or impaired function of this lysosomal enzyme. This defect results in a reduced degradation of substrates with terminally α-glycosidically bound galactose (mostly Globotriaosylceramide Gb3) and the subsequent accumulation of these metabolic products in lysosomes of virtually all cells of the human body.

As Fabry’s disease is an X-linked disorder hemizygous males are more frequently and more severely affected than females. Still, recent data has also revealed the presence of overt Fabry’s disease in females with heterozygous mutations in the *GLA* gene [1]. Here, clinical variation is more diverse compared to males. Some females are not affected at all, even at higher age, and other females have a full-blown Fabry phenotype.

Clinically, Fabry patients present with diverse symptoms, already starting during childhood and adolescence. These symptoms include hypohidrosis, acroparaesthesia, muscle pain, episodic diarrhea or abdominal pain as well as angiokeratomas and recurrent fever [1].

One of the most common causes of death in males is end-stage renal disease (ESRD), preceded by proteinuria, hematuria, hypertension and renal insufficiency. However, the pathophysiology resulting in ESRD is incompletely understood. Endothelial damage in Fabry patients can be dramatic and contributes to deterioration of renal function. Additionally, histological studies suggest that podocyte damage is involved [1], [4], [5]. Gb3 severely accumulates in these cells resulting in the development of focal and segmental glomerulosclerosis (FSGS). Podocytes are unique neuron-like glomerular cells that are highly differentiated. With their foot processes they form the slit diaphragm and thereby play a major role in the maintenance of the glomerular filtration barrier. Damage to podocytes results in proteinuria and in the histological presentation of glomerulosclerosis [6], [7], leading to ESRD. Podocytes only have a limited potential of self-renewal [6], a fact that could make them particularly susceptible to damage by lysosomal storage disorders.

It has recently been shown that podocytes can directly take up extracellular α-Gal A via mannose 6-phosphate/insulin-like growth II receptor as well as megalin and sortilin [8]. Furthermore, enzyme replacement therapy with exogenous administration of α-Gal A, that has been available for 10 years, may be helpful for treatment of renal disease [9], [10], [11], [12]. However, the mechanisms leading to podocyte damage in Fabry’s disease have not been studied yet. As mice differ from humans in their glomerular lipid metabolism this question cannot be addressed in the Fabry mouse model [13], [14].

To study podocyte dysfunction in Fabry’s disease we therefore generated a human cell culture model. In an established human podocyte cell line α-Gal A expression and activity was reduced by lentiviral transfer of small hairpin RNAs. The characterization of this cell line revealed a novel link between Fabry’s disease and autophagy pathways. This model may serve as a tool to generate new insights into the pathophysiology of glomerular impairment in Fabry’s disease.

## Materials and Methods

### Cell Culture and Transfections

HEK293T cells were cultured in DMEM supplemented with 10% fetal bovine serum (FBS). For transfection experiments, cells were grown to 60–80% confluence and transfected with plasmid DNA using a modified calcium phosphate method.

The conditionally immortalized human podocyte cell line has been developed by transfection with the temperature-sensitive *SV40 T*-gene as recently described [15]. Proliferating at a temperature of 33°C, these cells transform into a quiescent, differentiated phenotype after transfer to 37°C. Cells were cultured in standard RPMI media containing 10% fetal bovine serum (Biochrom, Berlin, Germany) and insulin-transferrin-sodium selenite supplement (Gibco, Karlsruhe Germany). For experiments cells were grown at 37°C for at least 14 days until they were differentiated showing an arborized morphology.

### Generation of Stable Cell Lines and Dual Luciferase Assay

shRNAs were designed based on the prediction of publicly available prediction programs. Different shRNAs were cloned into the transient micro-RNA expression vector pcDNA6.2-GW/emGFP/miR (Invitrogen, Carlsbad, CA, USA), which coexpresses the shRNA surrounded by miR-155–flanking sequences together with emGFP. To monitor the efficiency of shRNA-mediated knockdown, we created a luciferase reporter construct using psicheck2 (Promega, Mannheim, Germany) in which the coding sequence of human *GLA* was fused to the coding sequence of renilla reniformis luciferase as an artificial 3′ UTR. In addition to renilla reniformis luciferase, this construct expresses firefly luciferase for internal control. 50 ng of the reporter plasmid was co-transfected with 50 ng of the respective pcDNA6.2-GW/emGFP/miR shRNA construct into HEK293T cells in a 96-well format using lipofectamine 2000 (Invitrogen, Carlsbad, CA, USA) as a transfection reagent. Renilla reniformis luciferase and firefly luciferase activities were measured by a dual-luciferase reporter assay system (Promega, Mannheim, Germany) in a luminometer (Mithras LB940; Berthold) 24 h after transfection. Results represent renilla/firefly luciferase ratios from three independent experiments performed in triplicates. Error bars represent SEM. Selected shRNA and a scrambled negative control (co shRNA) were GATEWAY-cloned into pLenti6.2/V5/TO/Dest for stable lentiviral expression in human podocytes. Lentiviral gene transfer was performed using standard methods. Blasticidin was used for selection. shRNAs had the following sequences: shRNA 361∶5′-TGCTGTGCAGAGGTACTCATAACCTGGTTTTGGCCACTGACTGACCAGGTTATGTACCTCTGCA-3′; shRNA 459∶5′-TGCTGATAATTAGCTAGCTGGCGAATGTTTTGGCCACTGACTGACATTCGCCATAGCTAATTAT-3′; shRNA 726∶5′-TGCTGTTGAAAGGGCCACATATAAAGGTTTTGGCCACTGACTGACCTTTATATGGCCCTTTCAA-; shRNA 894∶5′-TGCTGTAACATATCTGGGTCATTCCAGTTTTGGCCACTGACTGACTGGAATGACAGATATGTTA-3′; co shRNA (scrambled shRNA): 5′-TGCTGAAATGTACTGCGCGTGGAGACGTTTTGGCCACTGACTGACGTCTCCACGCAGTACATTT -3′.

### Rapamycin and Bafilomycin A Treatment

Cells were incubated as indicated with 200 nM of rapamycin (Sigma-Aldrich, Taufkirchen, Germany) for 24 h or with 100 nM bafilomycin A (Sigma-Aldrich, Taufkirchen, Germany) for 1 h prior to preparation of cell lysates. Control cells were incubated with vehicle (DMSO) alone.

### Cloning, Antibodies and Plasmids, Quantification of Western Blot Analysis

The coding sequence of human *GLA* was PCR-amplified from a human podocyte library and cloned into a modified pcDNA6/FLAG expression vector (Invitrogen, Carlsbad, CA, USA). Antibodies and antisera were obtained from Sigma (St. Louis, MO, USA; anti-FLAG), Cell Signaling (Danvers, MA, USA; anti-phospho AKT (S473), anti-AKT, anti-phospho-mTOR (S2448), anti-mTOR, anti-phospho-p38 (Thr180/Tyr182), anti-p38, anti-phospho-p42/44 (Thr 202/Tyr204), anti-p42/44), BD (Heidelberg, Germany, anti-β-catenin), Developmental Studies Hybridoma Bank (University of Iowa, USA, anti-β-tubulin), Santa Cruz (Heidelberg, Germany, anti 14-3-3), and MBL (Woburn, MA, USA, anti-LC3).

Western Blot analysis were performed using standard techniques. Proteins were resolved by SDS-PAGE and visualized with enhanced chemiluminescence after incubation of the blots with the respective antibodies. For quantification of band intensity of western blot analysis individual bands of at least three independent experiments were quantified using ImageJ software. The intensity of every band is expressed as ratio of the corresponding loading control (β-tubulin, 14-3-3 or β-catenin). All three proteins used as loading control are abundantly expressed in podocytes and did not show regulation by *GLA* shRNAs. Error bars shown in the figures represent SEM.

### Quantitative RT-PCR

Human podocytes were transduced with the indicated shRNAs and differentiated at 37°C for at least 14 days. Cells were harvested in Qiazol (Qiagen, Hilden, Germany) and RNA was isolated according to the manufacturer’s protocol. Reverse transcription was performed with ABI’s HighCapacity cDNA Kit (Applied Biosystems, Darmstadt, Germany). SYBR Green quantitative real-time PCR was performed to evaluate mRNA levels, and *HPRT1* and untransduced human podocytes served as endogenous control. qPCR assays were considered valid only if untransduced podocytes and control shRNA did not differ more than a factor of 1.6. Primers had the following sequences: *GLA*: 5′ – TGGAAGGATGCAGGTTATGAG - 3′ (fp) and 5′ – CCCTAGCTTCAGTCCTTTGCT –3′; *HPRT1*∶5′ – TGACACTGGCAAAACAATGCA –3′ (fp) and 5′ – GGTCCTTTTCACCAGCAAGCT –3′. All qPCR experiments were performed on the ABI 7900HT System and repeated four times. Error bars shown in the figures represent SEM.

TaqMan assays were performed using biological triplicates. 20 ng cDNA were used as a template in a 20 ul reaction volume using the TaqMan GeneExpression Master Mix (LifeTechnologies). Cycling and detection were performed using an HT7900 qPCR cycler (Applied Biosystems). The following TaqMan assays were used in the experiments (all ordered from LifeTechnologies labeled with FAM): Hs00234508_m1 (MTOR), Hs00178289_m1 (AKT1), Hs01086102_m1 (AKT2), Hs00178533_m1 (AKT3). The following TaqMan assay was used as endogenous control (LifeTechnologies VIC labeled): Hs99999903_m1 (ACTB). The following primer pairs were used for SYBR green based qPCR reactions using PowerSybrGreen master mix (LifeTechnologies). Again 20 ng cDNA were used in a 20 ul reaction volume: PGK1 fp CTGTGGGGGTATTTGAATGG; rp CTTCCAGGAGCTCCAAACTG. qPCR analysis was performed using SDS RQ Manager and DataAssist 3.01 (Applied Biosystems) and employing both endogenous controls described above. Significance was calculated using a two-tailed Student's t-test. Error bars represent SEM.

### α–galactosidase A Activity Assay

Podocytes were collected from culture dishes and lysed in PBS by five cycles of freezing and thawing. α-gal A activity was fluorimetrically determined by incubating aliquots of the supernatant at 37°C with 4-methylumbelliferyl α-D-galactopyranoside as substrate (Sigma, Taufkirchen, Germany) as previously described [16]. For internal control, activity of β-gal was measured with 4-methylumbelliferyl β-D-galactopyranoside. Protein concentration was determined by Pierce BCA protein assay kit (Thermo Scientific, Rockford, IL, USA).

### Lipid Analysis

Approximately 10^7^ cells were homogenized in 1 ml of water using the Homogenisator Precellys 24 (Peqlab, Erlangen, Germany) at 6.500 rpm for 30 sec. The protein content was routinely determined using bicinchoninic acid. After addition of 5 ml of methanol and 2.5 ml of chloroform, lipids were extracted for 24 h at 37°C. Insoluble cell debris was separated by filtration, and the solvent was evaporated in a stream of nitrogen. Interfering glycerolipids were degraded by alkaline hydrolysis with 2.5 ml of 100 mM sodium hydroxide in methanol for 2 h at 37°C. After neutralization with 15 µl of acetic acid, the lipid extract was desalted by reversed phase C18 (RP-18) chromatography. Neutral and acidic lipids were then separated by anion exchange chromatography using DEAE-Sephadex [17].

The neutral lipid fraction was applied to 20×10 cm high performance thin layer chromatography (HPTLC) Silica Gel 60 plates (Merck, Darmstadt, Germany), which were pre-washed twice with chloroform/methanol 1∶1 (v/v) and air-dried for 30 min. For quantification of neutral glycosphingolipids, each lane of the TLC plate was loaded with the equivalent of 600 µg of protein. The TLC solvent system used was chloroform/methanol/water 70∶30:4 (v/v/v). For quantitative analytical TLC determination, increasing amounts of standard lipids (*Neutral Glycosphingolipid Mix* containing glucosylceramides, lactosylceramides, globotriaosylceramides, and globotetraosylceramides, BIOTREND, Cologne, Germany) were applied to the TLC plates in addition to the lipid samples. For detection of lipid bands, the TLC plates were sprayed with a phosphoric acid/copper sulfate reagent (15.6 g of CuSO_4_(H_2_O)_5_ and 9.4 ml of H_3_PO_4_ (85%, w/v) in 100 ml of water) and charred at 180°C for 10 min [18]. Lipid bands were then quantified by densitometry using the TLC-Scanner 3 (CAMAG, Berlin, Germany) at a wavelength of 595 nm.

### Immunofluorescence Staining of Human Podocytes

Human podocytes were seeded and cultured on collagen-coated glass slides. After washing with ice-cold PBS, cells were fixed using methanol at −20°C for 5 min, and permeabilized with 0.05% Triton X-100 in dPBS for 8 min at room temperature. Cells were washed three times with dPBS. After an initial blocking step in 5% normal donkey serum, immunofluorescence staining was performed with a murine monoclonal LC3B clone 2G6 antibody (Nanotools. Teningen, Germany; 1∶500) After washing, slides were incubated with DyLight 549 secondary antibody, washed again, mounted in a commercially available antifade kit containing DAPI (Invitrogen, Carlsbad, CA), and subjected to immunofluorescence microscopy (Axiovert 200 with Apotome-System, Zeiss, Jena, Germany).

### TUNEL Assay

DEAD END™ colorimetric TUNEL system (Promega, Mannheim, Germany) was used according to manufactures protocol and mounted in vectastain (Vector, Burlingame, USA). Assay analysis was performed in a blinded way and AxiovisionSoftware by Zeiss was used for quantification.

### Statistical Analysis

All experiments were performed in at least triplicates. Significance was calculated using a two-tailed Student’s t-test. Values <0.05 were considered statistically significant. Error bars represent SEM.

## Results

### Selection of Efficient shRNAs Targeting the *GLA gene*

**Figure 1:**
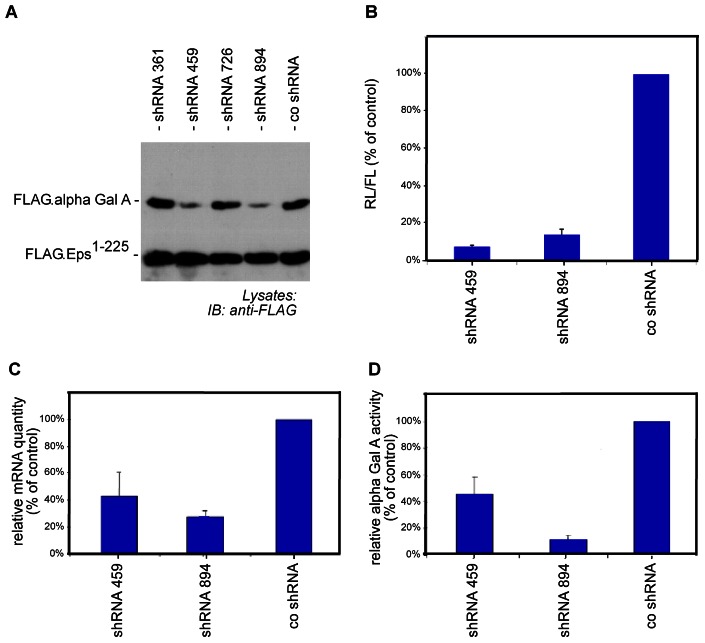
Validation of shRNA directed against the coding sequence of the *GLA* gene. (A) Different shRNAs were co-transfected with FLAG.α-galactosidase A cds in HEK293T cells. Co-transfection of FLAG.Eps^1–225^ served as transfection efficiency control. shRNAs 459 and 894 showed efficient reduction of α-Gal A protein levels as revealed by western blot analysis. (B) For the luciferase assay the coding sequence of *GLA* was fused with the coding sequence of renilla luciferase and cloned in a bicistronic expression plasmid containing the coding sequence of firefly luciferase (psiCheck2, Promega). This plasmid was co-transfected with the particular shRNAs. Renilla luciferase activity, normalized to firefly luciferase activity, was used to assay the efficiency of the shRNA-mediated knockdown of *GLA*. Scrambled shRNA was taken as a control. Experiments were performed in triplicates. Error bars represent SEM. (C) Human podocytes were transduced with either control shRNA or the indicated *GLA* shRNA. The specific knockdown of *GLA* was quantified by quantitative RT-PCR (n = 3). Error bars represent SEM. (D) α-Gal A activity in transduced human podocytes was assessed by an umbelliferone-based assay. ShRNA 894 showed a highly efficient reduction of α-Gal A activity (n = 3). Error bars represent SEM.

To study the glomerular pathogenesis of Fabry's disease we established a new cell culture model. Murine and human glomerular lipid metabolism differs greatly [14], which may explain why *Gla*-deficient mice do not show an overt glomerular phenotype [13]. We therefore opted for a knockdown approach in an established human podocyte cell model and generated various shRNAs targeting the coding sequence of human *GLA* gene. The efficiency of these shRNAs was first tested by co-transfection of the shRNA and a FLAG-tagged plasmid containing the α-Gal A coding sequence in HEK293T cells. These experiments revealed that shRNAs 459 and 894 could effectively reduce the expression levels of α-Gal A while the expression of a co-transfected control protein (F.Eps^1–225^) was unchanged (Fig. 1A).

In an independent approach we used a dual luciferase reporter assay to quantify the knockdown efficiency of the two selected shRNAs. We found that transfection of the selected shRNAs led to a knockdown of human α-Gal A of 92.4% for shRNA 459 and 86.2% for shRNA 894 (Fig. 1B).

### Generation and Characterization of Human *GLA* Knockdown Podocytes

We cloned the shRNAs into lentiviral gene transfer vectors to stably transduce an established human podocyte cell line [15]. The knockdown of *GLA* mRNA was confirmed by quantitative real-time PCR. The selected shRNA constructs showed a knockdown efficiency of 61% for shRNA 459 and 73% for shRNA 894 (Fig. 1C).

To functionally investigate whether this diminished amount of *GLA* mRNA resulted in a reduced activity of the of the α-Gal A enzyme we performed umbelliferone enzymatic assays. In this assay α-Gal A catalyzes the cleavage of 4-methylumbelliferyl-α-D-galactopyranoside leading to the release of fluorescent ß-methyl-umbelliferone that can be quantified. These assays confirmed that our knockdown approach led to a functional impairment of α-Gal A in human podocytes of 54% for shRNA 459 and 93% for shRNA 894 (Fig. 1D). Based on the finding that shRNA 894 was highly efficient whereas shRNA 459 did not show an extensive downregulation of α-Gal A activity we decided to continue our experiments with shRNA 894.

### Knockdown of *GLA* Induces Accumulation of Gb3 in Human Podocytes

**Figure 2:**
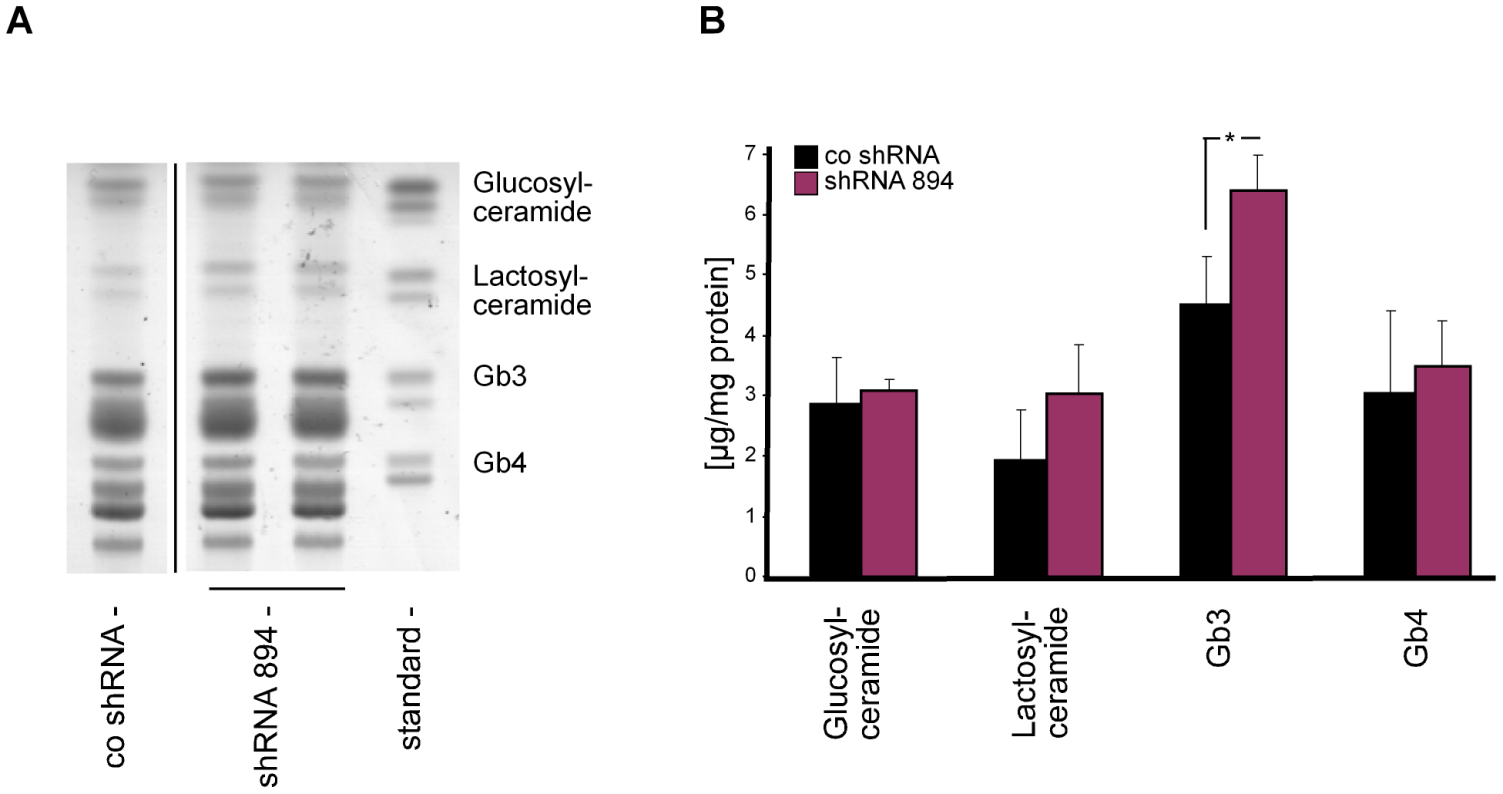
Accumulation of Gb3 in human *GLA* knockdown podocytes. (A) Analysis of lipid extracts from transduced podocyte cell lines by thin layer chromatography reveals the specific accumulation of Gb3 in human podocytes transduced with shRNA 894. (B) Quantification of lipid levels in the indicated cell lines (n = 3). Error bars represent SEM. * = p<0,05.

After having demonstrated reduced enzymatic activity of α-Gal A in our cellular model, we investigated whether this reduced enzyme activity leads to a cellular accumulation of Gb3. Analyses of lipid extracts from differentiated podocytes by thin layer chromatography (TLC) revealed that Gb3, but neither globotetraosylceramide (Gb4) nor glucosylceramide, significantly accumulated in our shRNA 894-transduced α-Gal A knockdown podocytes (Fig. 2). Significant accumulation of Gb3 demonstrated by TLC analysis suggested that shRNA 894-transduced human podocytes may serve as a model for the study of podocytes in human Fabry’s disease. These cells were used for subsequent analyses and compared to control shRNA-transduced cells at the same passage as a control.

### Fabry Podocytes Show Dysregulated Autophagy and Deficiencies in mTOR and AKT Signaling

**Figure 3:**
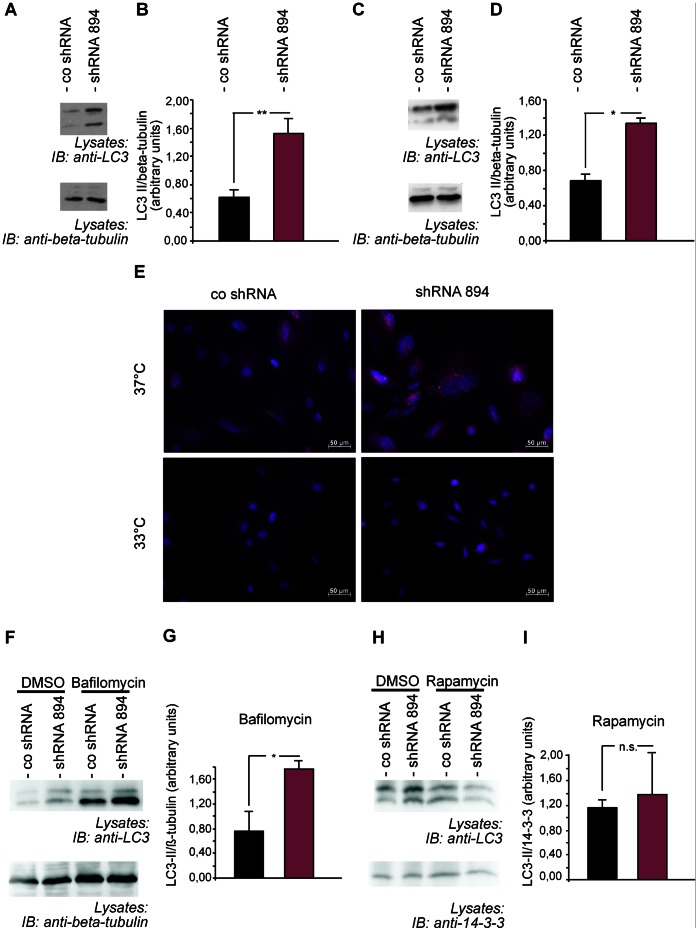
Fabry podocytes show an increase in LC3-II linking α-Gal A depletion to dysregulated autophagy. (A) Western blot of control podocytes (co shRNA) and α-Gal A knockdown podocytes (shRNA 894) revealing increased baseline expression of LC3-II in Fabry podocytes. (B) Quantification of baseline LC3-II expression in podocytes from three independent experiments. Error bars shown in the figures represent SEM. ** = p<0,01. (C) Increase of LC3-II in Fabry podocytes after stimulation of podocytes with IL-1ß in Fabry podocytes, shown by Western blot. (D) Quantification of LC3-II expression in podocytes after IL-1ß stimulation from three independent experiments. Error bars shown in the figures represent SEM. * = p<0,05. (E) Immunofluorescence staining of LC3 in control podocytes (co shRNA) and Fabry podocytes (shRNA 894). Differentiated, non proliferating Fabry podocytes grown at 37°C display a marked increase in autophagosomes compared to control cells. This difference was not observed in undifferentiated, proliferating cells grown at 33°C. (F) Bafilomycin treatment of control and Fabry podocytes shows an increase of LC3-II in both conditions compared to cells treated with vehicle (DMSO) alone. (G) Quantification of LC3-II in bafilomycin-treated control podocytes vs. Fabry podocytes. Error bars shown in the figures represent SEM of three independent experiments. * = p<0,05. (H) Treatment with rapamycin of control and Fabry podocytes shows an increase of LC3-II only in cells treated mit vehicle (DMSO). No difference was observed in rapamycin-treated podocytes (n = 4). (G) Quantification of LC3-II in rapamycin-treated control podocytes vs. Fabry podocytes. Error bars shown in the figures represent SEM of four independent experiments. n.s. = non significant.

To address the functional consequences of reduced α-Gal A activity and Gb3 accumulation in human podocytes we studied the cell biology of Fabry podocytes. We did not observe any major differences in the cytoskeletal structure or the formation of actin fibres (not shown). Recent compelling evidence suggests that the tight regulation of autophagy is especially important in podocytes [19], [20], [21], [22]. We therefore tested whether the lysosomal enzymatic deficiency and Gb3 accumulation may alter the activity of autophagy pathways in podocytes. Interestingly, α-Gal A deficient podocytes showed a marked upregulation of the autophagic marker microtubule-associated protein 1 light chain 3 (LC3-II), a mammalian homolog of the essential yeast autophagy protein Atg8 [23], both at baseline levels (Fig. 3 A, B) and after induction of stress kinase signaling with recombinant IL-1ß at 10 ng/ml for 1 h (Fig. 3 C, D). The effect on baseline LC3-II expression was independently confirmed by using shRNA 459. In accordance with our quantitative real time PCR data and the findings from the umbelliferone enzymatic assays the effect of shRNA 459 was less evident than the effect of shRNA 894 (Fig. S1).

**Figure 4:**
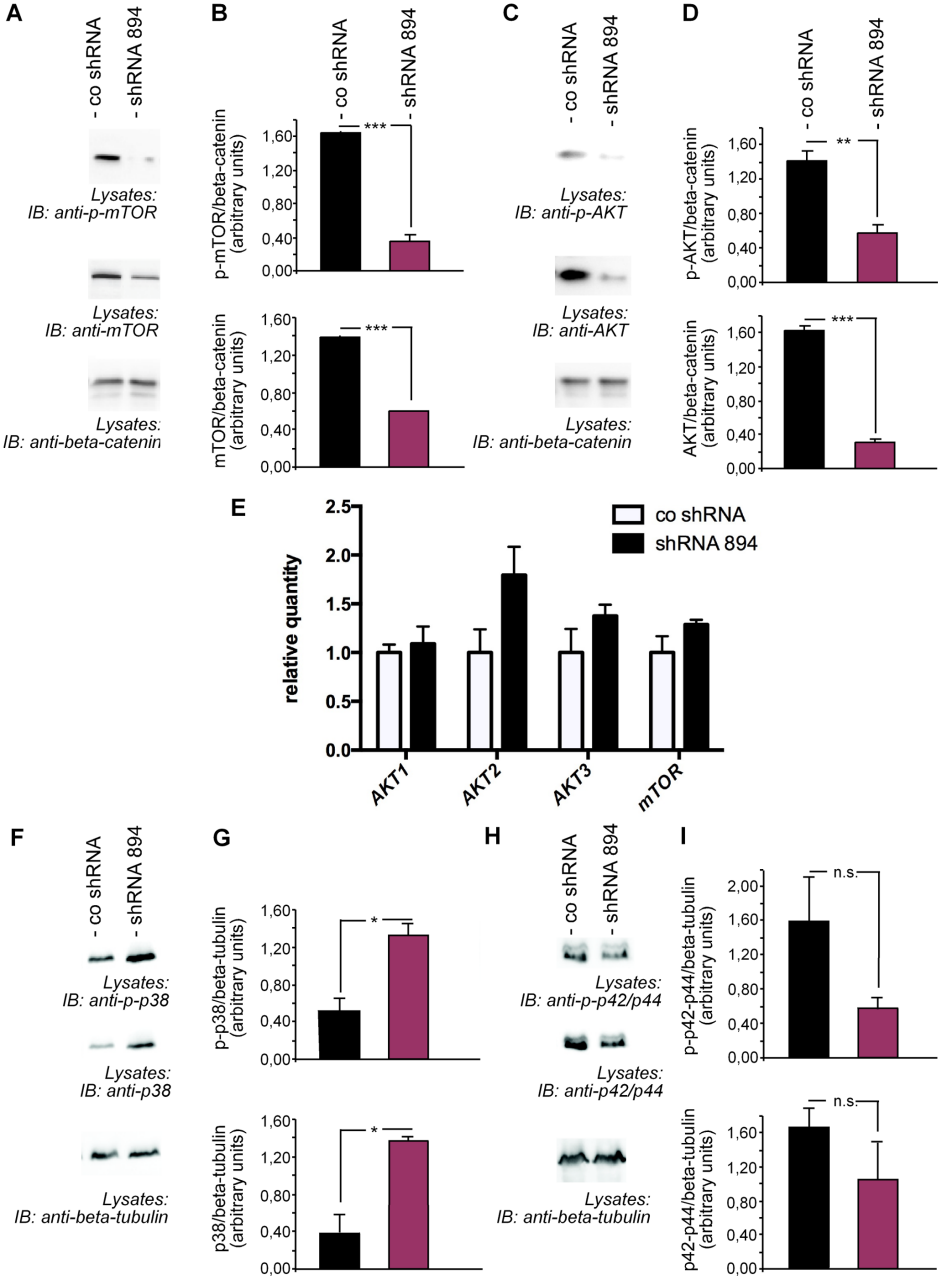
Fabry podocytes show defective AKT and mTOR signaling. (A) Western blot analysis revealed reduced phosphorylation of mTOR (serine 2448) and reduced protein expression of mTOR in Fabry podocytes. Cells were stimulated with IL-1ß for 60 minutes. (B) Quantification of phosphorylated and total mTOR expression in podocytes from three independent experiments. Error bars shown in the figures represent SEM. *** = p<0,001. (C) Decreased phosphorylation of AKT (serine 473) and reduced protein expression in Fabry podocytes after stimulation of podocytes with IL-1ß shown by Western blot analysis. (D) Quantification of phosphorylated and total AKT expression in podocytes from four independent experiments. Error bars shown in the figures represent SEM. ** = p<0,01, *** = p<0,001. (E) TaqMan assays show no significant difference in AKT1, AKT2, AKT3 or mTOR RNA levels of Fabry podocytes compared to control podocytes in three independent experiments. All AKT variants recognized by the AKT antibody used were tested. Error bars represent SEM. (F) Western blot analysis shows increased phosphorylation of p38 (Thr180/Tyr182) as well as increased protein expression of p38 in Il-1β-stimulated Fabry podocytes. (G) Quantification of phosphorylated and total p38 in Il-1β-stimulated podocytes from three independent experiments. Error bars represent SEM. (H) No significant change was observed in Western blot analysis of phosphorylated p42/44 (Thr 202/Tyr204) and of p42/44 protein levels in Il-1β-stimulated Fabry podocytes compared to control podocytes. (I) Quantification of phosphorylated and total p42/44 in Il-1β-stimulated podocytes from three independent experiments. Error bars represent SEM. n.s. = non significant.

**Figure 5:**
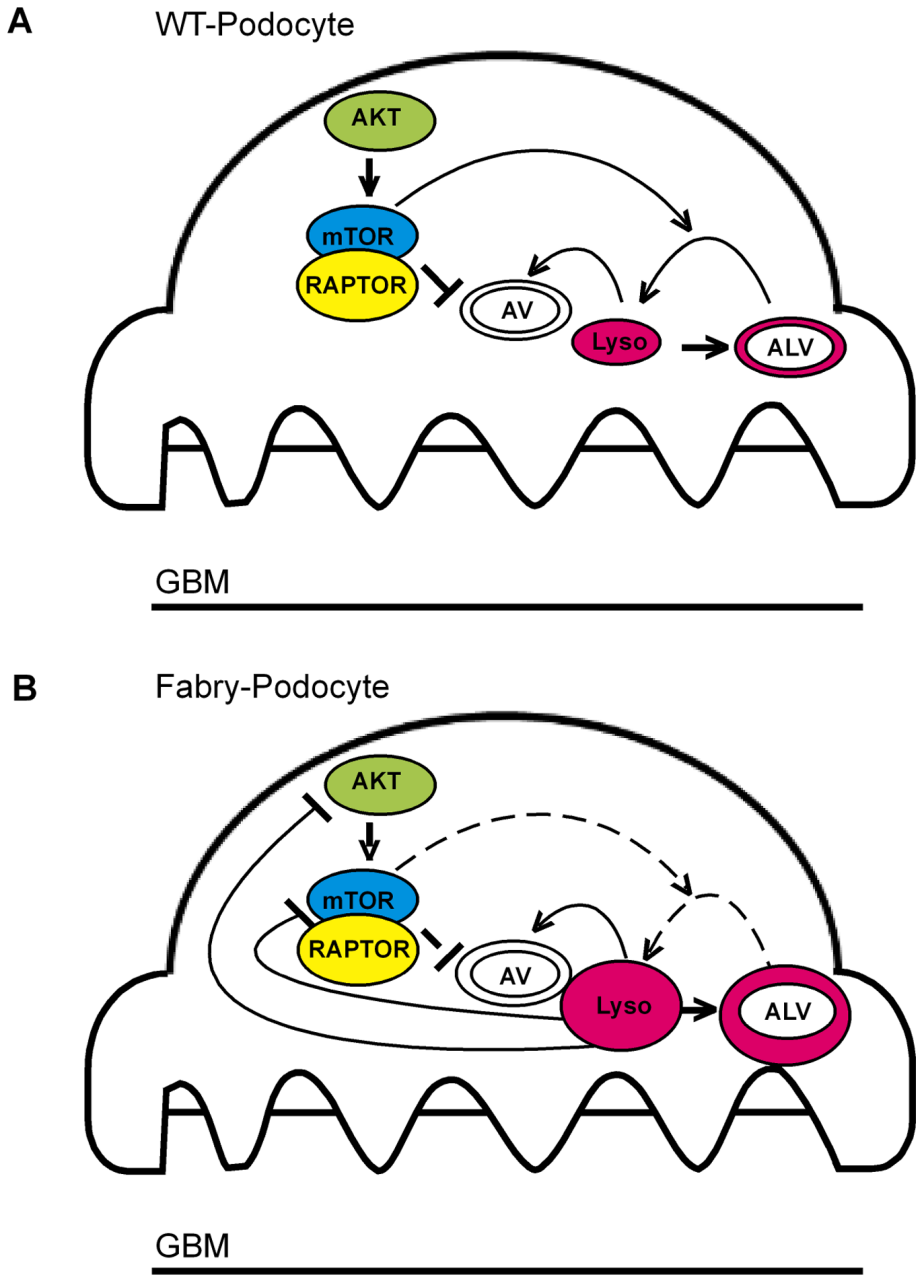
Model on the effect of α-Gal depletion in podocytes. mTOR negatively regulates the formation of autophagy vesicles and promotes the recovery of autophagosomes (AV) and lysosomes (Lys) from autophagolysosomes (ALV) in podocytes (continuous lines) (modified from [22]). In Fabry’s disease α-Gal A dysfunction leads to an accumulation of Gb3 in lysosomes, an increase in autophagosomes and furthermore dysregulates autophagy signaling (dashed lines) by an inhibition of mTOR and its upstream regulator AKT (continuous line).

Since it has been shown that the amount of LC3-II correlates well with the number of autophagosomes these data suggest that Fabry podocytes display grossly activated autophagic machinery. This hypothesis was supported by immunofluorescence stainings that revealed an increase of LC3-II-positive vesicles in α-Gal A deficient terminally differentiated podocytes (Fig. 3 E). In order to differentiate between an impaired maturation of autophagsomes on the one hand and an increase in formation of autophagosomes on the other hand we performed inhibitor experiments using the V-ATPase inhibitor bafilomycin A. Bafilomycin A blocks fusion of autophagosomes with lysosomes [24]. Treatment of cells with bafilomycin lead to an increase of LC3-II in both control and α-Gal A deficient podocytes suggesting a substantial change in autophagosome generation rather than an impaired maturation (Fig. 3 F, G). Mammalian target of rapamycin (mTOR) signaling has been identified as a critical regulator of autophagy and suppresses the formation of autophagic vesicles [23] Recent data also implicated podocyte mTOR signaling in the regulation of autophagosome and lysosome regeneration from autolysosomal vesicles [22]. To test whether a dysregulated mTOR activity may be responsible for the upregulation of autophagosomes in α-Gal A deficient podocytes we treated IL1-stimulated control and α-Gal A deficient podocytes with the mTOR inhibitor rapamycin (Fig. 3 H, I). Interestingly, we observed an increase in LC3-II only in control cells but not in α-Gal A deficient podocytes Therefore, we tested for mTOR activity by using phosphospecific antibodies. Phosphorylation of mTOR at serine 2448 increases mTOR activity. Activated Fabry podocytes showed less phosphorylation of mTOR indicating less activation of the mTOR pathway (Fig. 4 A, B). In addition, the deficient mTOR activity was accompanied by a marked downregulation of AKT activity (Fig. 4 C, D) suggesting that upstream autophagy signaling may be regulated in a feedback loop in podocytes. Activated AKT has previously been shown to increase mTOR signaling. Strikingly, AKT and mTOR expression levels were specifically downregulated in Fabry podocytes. To understand the underlying mechanism of AKT and mTOR regulation we performed TaqMan-based quantitative PCRs. All AKT variants that are recognized by the AKT antibody used in our experiments were tested. Interestingly, there were no significant changes in the mRNA expression of the studied genes (Fig. 4 E). To investigate whether the observed changes in AKT and mTOR expression were specific we expanded our experiments to further key signalling proteins that have been linked to autophagy. In contrast to mTOR and AKTthe mitogen activated stress kinase p38 was upregulated on the protein level and to a comparable degree on its phophorylation level (Fig. 4 F, G) whereas protein level and phosphorylation status of kinase p42/44 were unchanged (Fig. 4 H, I), thereby excluding a general reduction of protein levels in α-Gal A-deficient podocytes. Fitting to an impairment of AKT signalling a TUNEL assay points to a slightly decreased cell viability in knockdown cells (Fig. S2). These data suggest that dysregulated lysosomal protein degradation in human Fabry podocytes may directly affect AKT and mTOR stability and autophagy signaling (Fig. 5).

## Discussion

Dialysis-requiring chronic kidney disease is one of the major clinical problems in Fabry’s disease. While recent clinical work has convincingly addressed the value and efficiency of α-Gal A enzyme replacement therapy [9], [10], [11], [12], the underlying pathophysiology leading to the development of renal functional impairment in Fabry patients remains obscure. So far, little work has been undertaken to elucidate molecular mechanisms of renal disease development and progression in this lysosomal storage disorder.

During the last decade, multiple studies have confirmed that podocyte damage ultimately results in development of proteinuria, FSGS and ESRD. These studies highlight the fundamental role of podocytes for the functional and structural maintenance of the glomerular filtration barrier [7], [25]. In Fabry patients, proteinuria and FSGS are hallmarks of renal disease. Renal pathomorphology reveals that podocytes show the largest amount of glomerular lipid accumulation [26] in Fabry patients. The development of FSGS during the course of this lysosomal storage disease suggests involvement of terminally-differentiated podocytes in the pathogenesis of glomerular injury. Still, functional studies have been missing. This may partly be due to the problem that the α-Gal A knockout mouse model of this glycosphingolipid storage disorder does not show a glomerular phenotype [13]. This discrepancy between mouse and men could be explained by differences in local glycosphingolipid metabolism. Indeed, Gb3 accumulation has not been found in murine glomeruli [27], [28], [29], furthermore suggesting that the accumulation of Gb3 in human podocytes may not only be due to uptake of Gb3 from the serum into podocytes but also due to endogenous accumulation. The fact that podocytes have a very limited potential of self-renewal makes them prone for life-long deposition of sphingolipids and subsequent damage by this “debris” [6].

We used RNAi technique in combination with lentiviral gene transfer to generate a human cellular model of Fabry’s disease in an established human podocyte cell line. Our data confirm the efficiency of the chosen knockdown approach in multiple ways, on mRNA, protein and enzymatic functional level. Furthermore, we show the accumulation of Gb3 in our knockdown cells. Already, these data have various important implications as they confirm the functional relevance of endogenous α-Gal A in human podocytes. A recent publication was not able to detect α-Gal A protein in human kidney samples [30]. However, the group relied on immunohistochemistry and may therefore have missed quantitatively low expression levels of α-Gal A that would still be functionally relevant. In our cellular model we did detect expression of α-Gal A in podocytes and furthermore show that its impairment has functional consequences within the cell. Importantly, our data suggest that Gb3 content in human podocytes is, at least partly, caused by endogenous accumulation of Gb3 and not only by podocytic Gb3 uptake.

The accumulation of glycosphingolipids in podocytes could have various implications. Lipid-protein interactions in podocytes play an important role in intracellular signal transduction of podocytes [31]. In addition to the established role of slit diaphragm signaling for podoycte cell survival and polarity [32], recent data have pointed to the significance of autophagy-associated signaling in the development of glomerulopathies [19], [20], [21], [22]. Autophagy enables the cell to have access to nutrients in situations of stress or starvation. Under these conditions intracellular material is degraded in a lysosome-dependent mechanism, and autophagy serves as an intracellular recycling system [23]. For this mechanism an isolation membrane engulfs intracellular targets to become an autophagosome containing the LC3-II isoform of the essential autophagy protein LC3. The autophagosome then fuses with a lysosome to form a so-called autophagolysosome, which contains damaged and dysfunctional organelles like mitochondria. However, autophagosomes and lysosomes can also reform from autophagolysosomes [22], [23].

A link between lysosomal storage disorders and autophagy dysregulation has previously been suggested [33]. Activation of autophagy occurs in different lysosomal storage disorders [34]. Cells of murine models of the lysosomal storage disease multiple sulfatase deficiency contain elevated LC3-II levels and show an accumulation of autophagosomes [35]. It has also been suggested that fusion of autophagosomes with lysosomes may be impaired in these mice. For Fabry’s disease a recent publication revealed an upregulation of LC3-II in human Fabry skin fibroblasts and in kidney biopsies, but functional data on this link remains sparse [36]. Sphingolipids like ceramide can regulate autophagy in diverse ways with ceramide being an activator of autophagy [37]. Interestingly, mTOR in podocytes has been linked to deactivation of autophagy but also to the efficient regeneration of autophagosomes and lysosomes from autolysosomal vesicles [22]. It has been speculated that mitochondrial dysfunction or accumulation of acidophilic autophagolysosomes containing damaged organelles in postmitotic cells with suppressed lysosomal activity can contribute to cell damage in neurons and podocytes [22], [33].

Our data suggest basal accumulation of autophagosomes in Fabry podocytes. After induction of stress kinase signaling by IL1ß, Fabry podocytes showed a decreased activity of the AKT-mTOR pathway. As mTOR activation is known to decrease autophagy this suggests that autophagy may be overactive in Fabry podocytes. Indeed, LC3-II levels continued to be elevated after stress induction. Overactive autophagy may be a contributor to podocyte damage in Fabry’s disease. In our experimental setting we did not find evidence for disturbed autophagic maturation in Fabry podocytes. Our data fit nicely to the recent publication of a murine podocyte-specific knockout of mTOR that exhibited accumulation of LC3, autophagosomes, autophagolysosomal vesicles, and damaged mitochondria in this highly differentiated cell type [22]. The described mouse model showed early heavy proteinuria. Furthermore, in human podocytes treated with an mTOR inhibitor accumulation of autophagosomes and autophagolysosomes was detected [22]. As expected we also observed an increase in LC3-II levels in our control podocytes upon mTOR inhibition. This increase was abolished in α-Gal A deficient podocytes, potentially due to decreased endogenous mTOR acitivity in these cells. Podocyte damage and loss have been shown to result in FSGS, the final histological pattern of injury in Fabry patients.

In summary, we established a human podocyte model of Fabry’s disease by stable depletion of *GLA* mRNA. These cells show reduced levels of enzyme activity and an accumulation of Gb3. Functionally, Gb3 accumulation was associated with an increase in autophagosomes and a decrease of mTOR and AKT signaling cascades might presdispose Fabry podocytes to further cellular damage. This cell line will serve as a promising tool for ongoing studies on the pathomechanisms of glomerular damage in Fabry’s disease.

## Supporting Information

Figure S1 **Confirmation of the effect of** ***GLA*** **knockdown on LC3-II expression with shRNA 459.** (A) Western blot analysis of control podocytes (co shRNA) and α-Gal A knockdown podocytes (shRNA 459) revealing increased baseline expression of LC3-II in Fabry podocytes. (B) Quantification of baseline LC3-II expression in podocytes from five independent experiments. Error bars shown in the figures represent SEM. ** = p<0,01.
(TIF)

Figure S2 **Evaluation of cell viability by TUNEL assay.** Cells were cultured in equal density. Cell viability was blindly assessed by TUNEL assay in proliferating and differentiated podocytes.
(TIF)
