# Supplementary figures and images for: Dysregulated Autophagy Contributes to Podocyte Damage in Fabry’s Disease
Source: PLoS One. 2013 May 17;8(5):e63506. doi: 10.1371/journal.pone.0063506 (PMC3656911; doi:10.1371/journal.pone.0063506)

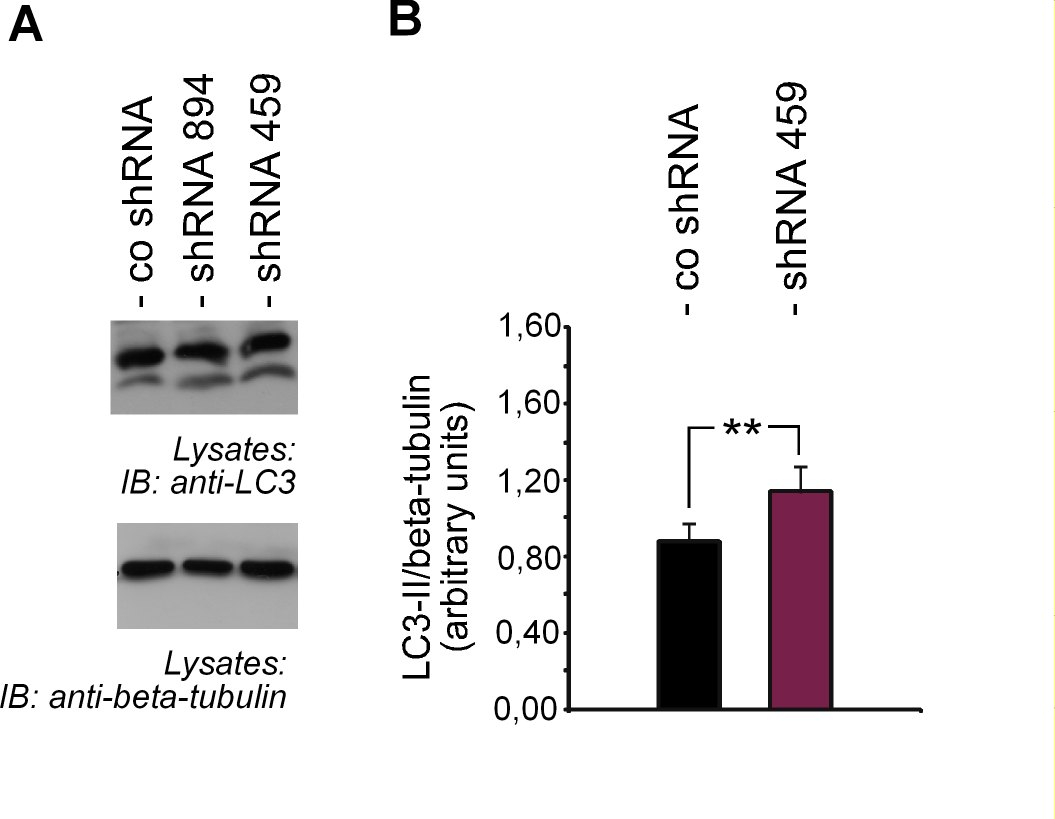

Supplement: Figure S1 — Confirmation of the effect of GLA knockdown on LC3-II expression with shRNA 459. (A) Western blot analysis of control podocytes (co shRNA) and α-Gal A knockdown podocytes (shRNA 459) revealing increased baseline expression of LC3-II in Fabry podocytes. (B) Quantification of baseline LC3-II expression in podocytes from five independent experiments. Error bars shown in the figures represent SEM. ** = p<0,01. (TIF) [file pone.0063506.s001.tif]

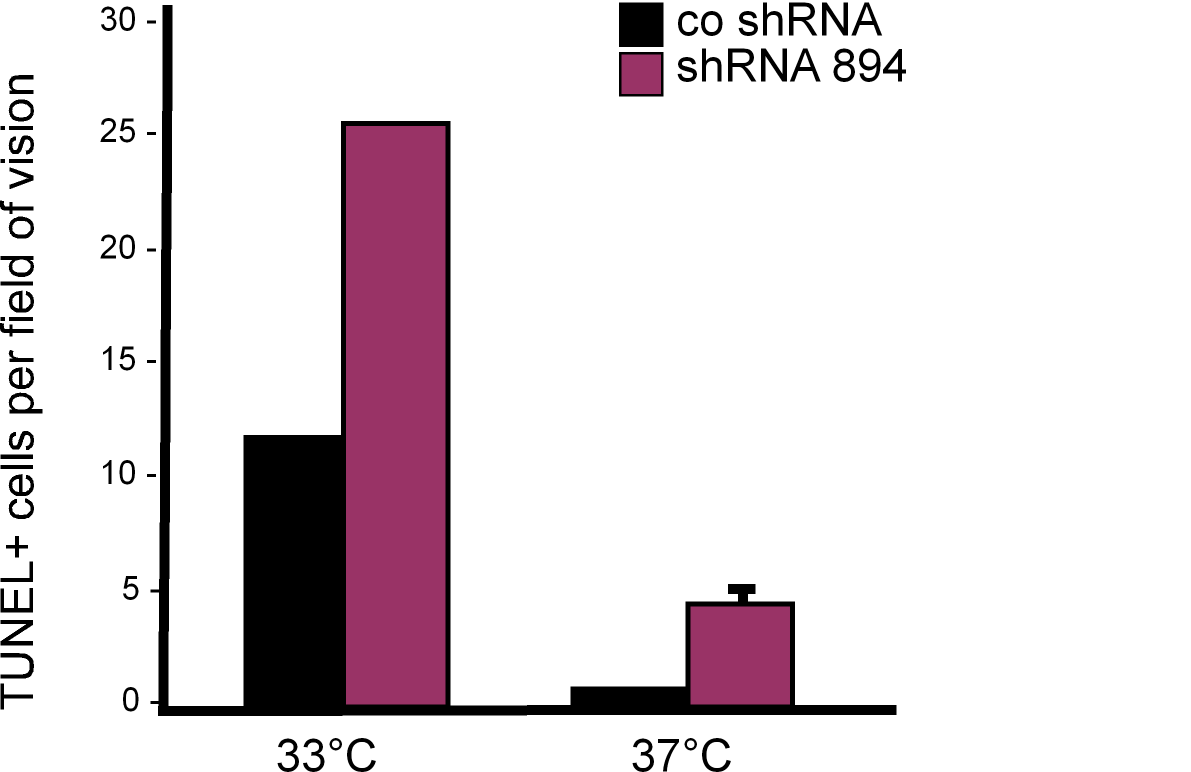

Supplement: Figure S2 — Evaluation of cell viability by TUNEL assay. Cells were cultured in equal density. Cell viability was blindly assessed by TUNEL assay in proliferating and differentiated podocytes. (TIF) [file pone.0063506.s002.tif]
